# Liquid Crystal Droplet-Based Biosensors: Promising for Point-of-Care Testing

**DOI:** 10.3390/bios12090758

**Published:** 2022-09-15

**Authors:** Ruwen Xie, Na Li, Zunhua Li, Jinrong Chen, Kaixuan Li, Qiang He, Lishang Liu, Shusheng Zhang

**Affiliations:** 1 Shandong Provincial Key Laboratory of Detection Technology for Tumor Markers, Linyi University, Linyi 276005, China; 2 College of Chemistry and Bioengineering, Hunan University of Science and Engineering, Yongzhou 425100, China

**Keywords:** LC droplets, biosensing, POC, single-cell monitoring, enzyme sensors, clinical applications

## Abstract

The development of biosensing platforms has been impressively accelerated by advancements in liquid crystal (LC) technology. High response rate, easy operation, and good stability of the LC droplet-based biosensors are all benefits of the long-range order of LC molecules. Bioprobes emerged when LC droplets were combined with biotechnology, and these bioprobes are used extensively for disease diagnosis, food safety, and environmental monitoring. The LC droplet biosensors have high sensitivity and excellent selectivity, making them an attractive tool for the label-free, economical, and real-time detection of different targets. Portable devices work well as the accessory kits for LC droplet-based biosensors to make them easier to use by anyone for on-site monitoring of targets. Herein, we offer a review of the latest developments in the design of LC droplet-based biosensors for qualitative target monitoring and quantitative target analysis.

## 1. Introduction

Liquid crystal (LC) is a kind of substance whose morphology is between crystal and liquid, which has not only the anisotropy of crystal but also the fluidity of liquid. The anomalous behavior of cholesterol benzoate during melting was observed by Austrian biologist Friedrich Reinitzer in 1888, and this substance was called “crystalline liquid” by Otto Lehman and confirmed as a defined state of matter by French crystallographer Georges Friedel in 1922 [1]. According to the different physical conditions and components, LCs are generally divided into thermotropics and lyotropics. Thermotropic LCs are typically formed by pure organic compounds in a specific temperature range. Usually, with an increase in temperature, the morphology of the substance changes from a crystal state to turbid liquid and then from turbid liquid to transparent liquid. The critical temperature between these three forms is the melting point and the clear point. The state between these two degrees is thermotropic LC, which has birefringence and dielectric anisotropy [2,3]. Thermotropic LCs can be classified into smectic LCs (SLCs), nematic LCs (NLCs), and cholesteric LCs (CLCs) according to the arrangement order of LC molecules [4]. Lyotropic LCs generally form when one or more solute molecules are in a specific concentration range in the solvent, which is usually water or other polar solvents.

The molecules of SLCs are mostly in the form of rods or strips, arranged in parallel with each other to form a layer structure, and the intermolecular action of each layer is weak. The LC molecules can slide inside the layer, but cannot move up and down between adjacent layers, and there is a definite distance between layers, which makes it easy for a relative motion to occur [5]. The nematic liquid crystals have molecules that are parallel to one another and maintain their one-dimensional arrangement without delamination. Although the nematic phase lacks positional order, it possesses great orientational order. Nematic phases can be calamitic or discotic depending on the structure of the molecule. This directional orientation results in anisotropy, which affects the nematic phase’s birefringence, magnetic susceptibility, and dynamic behavior [6]. The CLCs have flat molecules. They exhibit chirality and have a helical shape between the layers and molecules that are parallel to one another inside the layers [7]. The CLCs can be prepared by doping non-chiral nematic LCs with chiral dopants or by incorporating chiral centers in the nematic structure. The pitch length of the helix can be simply changed by using external stimuli, such as temperature, chiral doping concentration, and the intensity of external electric and magnetic fields, which is one of the advantages of doped CLC [8].

The construction of a LC droplet sensor is mainly based on LC’s long-range order and birefringence. The key to a LC droplet sensor is to design a modified sensitive surface with specific recognition target molecules. Under the action of the modified molecule, the LC molecules can arrange themselves in an orderly manner. However, this order will be disrupted when the target analyte is injected. In comparison to the LC molecule and the modified molecule, the force between the target molecule and the modified molecule is greater. For instance, through ligand-receptor interaction [9], hydrophobic interaction [10,11], and so on, which weakens the anchoring effect of the sensitive surface to the droplet, thus changing the order of the LC molecules. The refraction of light by the LC droplet is affected, resulting in changes in the brightness and color of the sensor under polarized optical microscopy (POM), thus realizing the detection of target molecules [12]. When the non-target material is added to the sensor, the modified sensitive molecules do not interact with them, the LC molecules maintain the original orientation; that is, it cannot produce a response signal. The arrangement of LC molecules is closely related to the existence of the sensitive interface, which can respond rapidly to the changes in the external environment and has a unique optical amplification effect. The response signal can be obtained effectively and efficiently with the help of a POM [13]. Today, non-invasive and point-of-care platforms that give real-time response and low detection limits are important for healthcare monitoring and clinical application [14,15,16,17,18]. The LCs are an effective sensing component that gives a convenient, cost-effective, and easy-to-read response platform.

Due to the ease with which stimuli-sensitive director configuration transitions may be implemented in flat geometries [19,20], in spherical geometries, i.e., droplets [21,22,23], and in shells [24,25], using LCs to identify chemical and biological species has become very common [26]. They can achieve the same or even better results than other sensing technologies [27,28,29]. Depending on how the orientation at the LC surface affects the structure throughout the droplet, the geometric confinements of the LC droplets can take on various forms. Because of their large specific surface area, various thermodynamic stability defects, and rich phase morphology texture, many researchers are interested in LC droplets. It is known that NLC droplets have a bipolar form and a radial form, with two boojums at the droplet’s opposing poles and a single point-of-defect in the center, respectively [30]. In contrast to NLC droplets, the helical molecular arrangement is an additional characteristic of CLC droplets. If CLC droplets are only affected by spherical geometry with tangential surface anchoring conditions, they show a Frank–Pryce spherulite pattern with concentric circles [31]. Recent studies have found that short-spaced CLC droplets show central spot reflection at CLC photonic bandgap (PBG) wavelengths in a planar anchoring environment. Still, in homeotropic circumstances, they show spots that seem like flashing lights [32]. In a word, the alignment of LC droplets can be precisely controlled under various conditions, making them an ideal material for various applications [33,34].

Currently, a significant portion of detector readouts relies on bared-eye examination of the optical appearance using POM [35,36]. The reaction process is challenging to quantify and accurately characterize in these sensing modalities, limiting the further development of the LC sensing device in real-time monitoring. In order to achieve real-time quantitative monitoring of the analyte reaction process and to overcome these limitations [37,38,39], many groups use whispering gallery mode (WGM) lasers in LC droplets [40,41,42]. When light is incident from a light-dense medium to a light-sparse medium and the angle of incidence is large enough, total reflection can happen at the intersection of the two media, producing an optical WGM at the surface of the high-refractive index medium. This phenomenon is analogous to the reflection of sound waves in the walls of a cloister. The light will be increased and bound in the cavity, generating the so-called WGM resonance [43]. Due to the flawless spherical structure and incredibly smooth interface, LC droplets make an excellent optical microcavity for WGM lasing [44,45,46]. In theory, excitation light from a gain medium undergoes multiple total internal reflections at the optical microcavity interface before being amplified by constructive interference [47]. The resonance frequency of the microcavity depends on the distribution of the refractive index along the optical path. The change of refractive index, in turn, can be detected by the WGM spectra as a resonance frequency shift. The use of WGM lasing in LC microdroplets is expected to become a promising tool in the field of biosensing due to the unique properties of LC materials.

Some advantages of this method include the following: (i) WGM lasing can convert the biochemical reaction process into a spectral response in real-time, providing more accurate and quantitative information; (ii) the kind and strength of the local molecules anchoring at the LC/water surface strongly influence the overall arrangement of LC molecules in microdroplets, hence the WGM resonance spectra will catch any minute changes in the anchoring state of LC molecules in real-time; (iii) the LC microdroplets’ high surface-area-to-volume ratio enables complete molecular reactivity at the LC/water interface and enhances the detection limit [48].

Compared with other traditional methods [49,50,51,52,53], a complete analysis is offered here, including a focus on more recent developments in the use of LC droplets in the design of biosensors for the on-site and real-time monitoring of various targets. Being used as a case study, a prospective viewpoint for developing innovative LC droplet-based biosensing test kits for point-of-care diagnosis may be obtained from this review.

## 2. Preparation of LC Droplets

The LC droplet method is a valuable technique for creating biosensors. The sensing mechanism is that the adsorption of target molecules on the droplet interface will first cause changes in the molecules on the LC droplet’s surface and then cause changes in the molecular arrangement inside the droplet, thus affecting the optical morphology of the LC droplets. As LC droplets have a large specific surface area, a variety of thermodynamically stable defects, and a rich phase structure, they have the advantages of requiring a small number of detection samples, a wide variety of detection samples, and high detection sensitivity [54].

### 2.1. Emulsion Method

**Figure 1:**
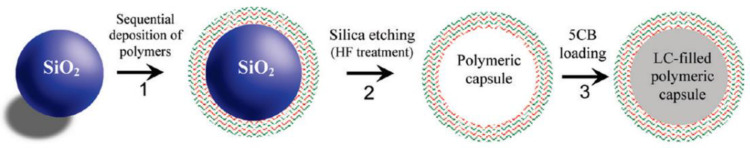
Schematic illustration of the procedure used to prepare LC droplets of predetermined sizes within polymeric multilayer shells. Reprinted with permission from ref. [55] Copyright 2009 American Chemical Society.

Researchers have built LC sensing substrates using the emulsion method, one of the principal methods of creating LC droplets. Using this technique, they studied the response signals of numerous molecules at the droplet interface. For instance, Gupta’s group [55] reported a silica template that was disseminated in an aqueous solution coated with polymers, such as poly-4-benzenesulfonic acid sodium (PSS) and polyallylamine hydrochloride (PAH), layer by layer. After the polymeric coating had been applied, the silica core was etched, and the resultant capsule was filled with low molecular weight LC (Figure 1). For independent control of LC droplet size and interfacial chemistry, the choice of silica template size and polymeric shell layer served as the foundation.

For early diagnosis and treatment, it is essential to design readout devices that are straightforward, sensitive, quick, and affordable [56,57,58,59,60]. Other emulsion preparation methods created thus far included photopolymerization, ultrasonication, shearing of droplets and subsequent crystallization fractionation, droplet breakoff in a coflowing stream, and dispersion polymerization, in addition to the conventional methods [61,62,63,64]. The preparation of emulsion droplets using these techniques is booming, but precise droplet size control is difficult. Additionally, because of the mobility of the droplets, it is challenging to gauge the response time of LCs as a sensing platform, which limits their use in real-time monitoring, analytical chemistry, and biomolecular applications. The preparation of emulsion droplets has been enhanced by several groups in an effort to address the issues. To obtain monodisperse LC droplets of uniform size, Sivakumar et al. [65] used silica as a template dispersed in solution assembled layers of hydrogen-bonded poly(methacrylic acid) (PMA) and poly(vinylpyrrollidone) (PVPON). Then silica was selectively etched off the template by hydrofluoric acid (HF) to fill the LC molecules into the cavity formed by the polyelectrolyte multilayer (PEM) film. The polyelectrolyte membrane dictated the chemical characteristics at the interface, and the size of the LC droplets generated by this approach may be adjusted by changing the size of the silica template.

**Figure 2:**
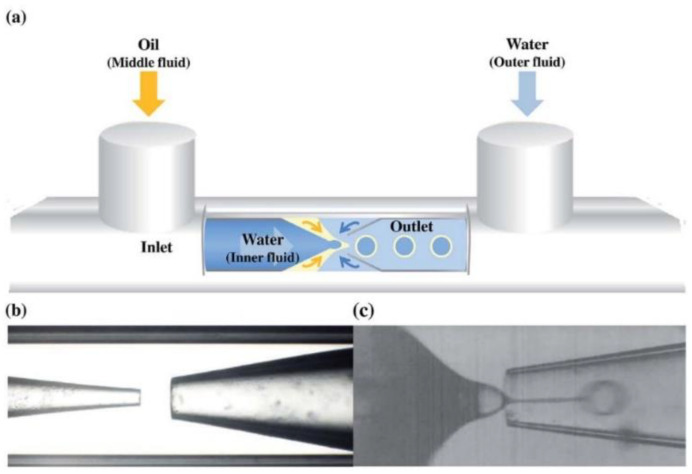
(**a**) Schematic of the capillary microfluidic device combining co-flow and flow-focusing geometries. Photographic images of the microfluidic capillary devices were used in the (**b**) absence and (**c**) presence of fluids. Reprinted with permission from ref. [67]. Copyright 2016 Royal Society of Chemistry.

In recent years, microfluidic technology [66] has also been applied to prepare LC droplets, providing a new method for obtaining uniform LC droplets. For example, Park’s group [67] used a microfluidic device to prepare monodisperse LC droplets modified by amphiphilic block copolymer and obtained droplets of various sizes by controlling flow rate (Figure 2). To break through the limitations of droplet mobility in real-time monitoring, analytical chemistry, and biomolecular applications, Fang et al. [68] used the principle that chitosan can form a gel rapidly after adding silver ions to embed LC droplets into chitosan-surfactant gel films to detect biomolecules. By this method, the advantage of the sensitive response of LC droplets is retained, and the real-time monitoring of the target detection substance can be realized, which provides a broad application prospect for the development of LC droplet sensors.

Many different types of biomolecules can be detected by LC droplets created using the emulsion approach, such as lipids, charged macromolecules, bacteria, viruses, and proteins [69,70,71]. The molecular arrangement in LC droplets is susceptible to the change in droplet size, so the optical morphology of LC can be changed by adjusting the droplet size. Then the target analyte can be detected, which provides a new detection method for LC sensors.

### 2.2. LC Droplet Pattern Method

**Figure 3:**
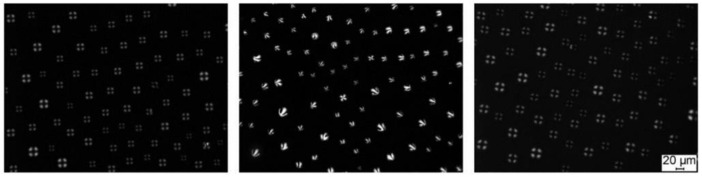
Polarized light microscopy images of surface-anchored LC droplet patterns in a different solution. Reprinted with permission from ref. [73]. Copyright 2014 John Wiley and Sons. The scale bar is 20 µm.

The LC droplet-based sensors have been developed using emulsion methods, although these technologies have certain drawbacks. For instance, droplets have a low degree of stability and a propensity to aggregate. These restrictions are thought to be solved by LC droplet pattern-based sensors. Two distinct LC droplet patterns spontaneously formed at the micrometer scale on solid surfaces offered the substantial potential for sensing applications. By adding LC dispersed organic solvents to the slides, researchers could essentially identify one-dimensional and two-dimensional LC droplet patterns with two unique optical textures. With large surface area, the surface anchored LC droplet may be used as a wide range of chemical and biosensors with higher sensitivity in water and gas environments. Jang’s group [72,73] developed the LC droplet pattern method for LC droplet preparation. Anhydrous ethanol and n-heptane were used to dissolve the LC 4-cyano-4′-pentylbiphenyl (5CB), mixed evenly by ultrasonic technique, and then added to Piraha washing solution and octyltrichlorosilane (OTS) treated glass substrate. After the organic solvent was evaporated, two different polarizing morphology (fan-shaped and cross-shaped) were obtained (Figure 3).

Han et al. [74] reported the method of a unique monodisperse LC droplet-based polydimethylsiloxane (PDMS) microchip with flow focusing components and hydrodynamics-based microstructures for the production and capture of monodisperse LC droplets, respectively. This LC droplet-based microchip had a set number of monodisperse LC droplets within, and by keeping an eye on how they collapse under POM, it could do real-time measurement with little sample consumption. Inkjet printing was employed by Yang’s group [75] to create consistent LC droplets that ranged in diameter from 35 to 136 μm and could be created by printing many times in the same location.

The findings mentioned above demonstrate that the LC droplet pattern method has considerable potential for the creation of straightforward, reliable, and adaptable sensors for the quick, accurate, and real-time detection of biomarkers. 

## 3. Overview of LC Droplet-Based Biosensors for POC Diagnosis of Diverse Targets

### 3.1. Bile Acid

Bile acids (BA) are significant metabolites that are essential for the emulsification and digestion of fats and lipolytic vitamins. Excessive BA production is connected to liver and intestinal disorders, however. As a result, diagnosing liver and intestine illnesses has traditionally relied on the study of BAs in various bodily fluids, including blood, liver, gallbladder fluid, and urine [76,77,78]. Cholic acid (CA) is a primary BA which comprises 31% of the total BAs produced in the liver. Secondary BAs include deoxycholic acid (DCA) and lithocholic acid (LCA). The correlation of liver and intestinal diseases with CA is much stronger than that of other BAs [79].

Host-guest recognition was used to design LC droplet-based sensors. Deng et al. [80] have reported the selective detection of CA by β-cyclodextrin (β-CD)/tetradecyl trimethylammonium bromide (C_14_TAB) complex coated with 5CB droplets. The β-CD was a naturally existing host molecule with a hydrophobic cavity that selectively recognized CA and formed an inclusion complex with a high equilibrium binding constant of 1:1. The CA displaced C_14_TAB from the cavity of β-CD, resulted in a conformational shift of the LC droplets.

**Figure 4:**
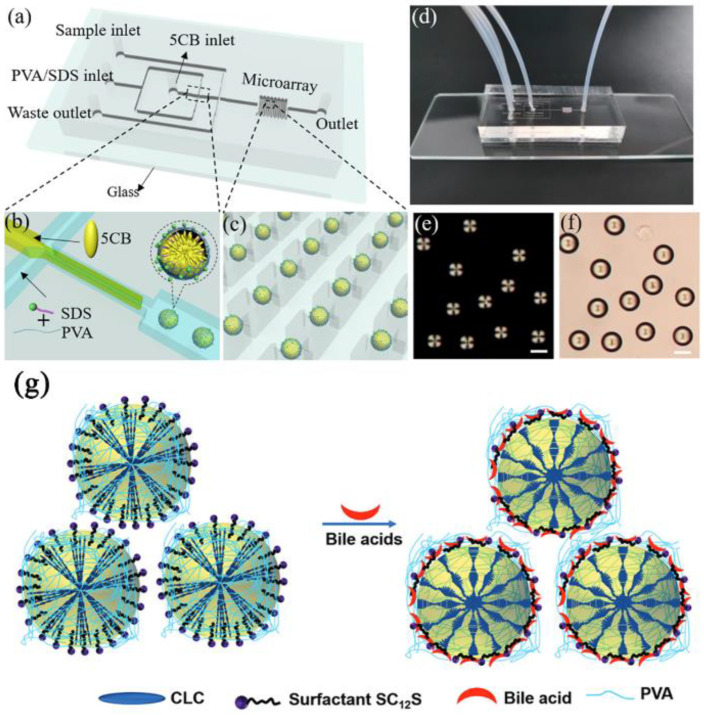
Schematic illustration of (**a**) the microchip to generate monodisperse 5CB droplets, (**b**) flow-focusing element, and (**c**) entrapment of the 5CB droplets in the microstructure. (**d**) Photograph of the microchip. (**e**) The POM and (**f**) bright-field images of the 5CB droplets. Reprinted with permission from ref. [74]. Copyright 2020 Elsevier. The scale bar is 50 µm. (**g**) Schematic illustration of the director configuration transition of PVA/SC_12_S-stabilized CLC droplets from homeotropic to planar, triggered by surfactant–bile acid interactions at the surface of the dispersed CLC droplets. Reprinted with permission from ref. [86]. Copyright 2021 Royal Society of Chemistry.

Detection by competitive adsorption was also a common method [81,82,83]. To detect LCA, Fang’s group [84] changed the interface of 5CB droplets distributed in an aqueous solution by adsorbing surfactants on the 5CB/aqueous interface. The detection limit of LCA is at the micromolar level. The lower limit of detection of surfactant stabilized 5CB droplets may be changed in the range of 10~70 µM by altering the chain length of the surfactant. A sensitive CA biosensor based on 5CB droplets in phosphate-buffered saline (PBS) was described by Niu et al. [85]. The radial-bipolar transition of the 5CB droplet was triggered when CA competed with sodium dodecyl sulfate (SDS) which loaded on the droplet’s surface. Their LC droplet sensor was quick, easy, and inexpensive. The detection limit of this method was 5 mΜ. Han et al. [74] reported a novel PDMS microchip based on the monodisperse droplet, wherein a single microfluidic device was used to create an in situ monodisperse droplets and capture them (Figure 4a–d). They could detect BAs quantitatively and quickly (<4 min) in real-time with little sample consumption (~1.5 μL) by observing the form of the LC droplets under a POM (Figure 4e,f). The detection limits for CA and DCA were 10 μM and 1 μM. A novel and highly sensitive biosensor for BAs (CA and DCA) based on polyvinyl alcohol (PVA)/SC_12_S stabilized CLC droplets was reported by Gollapelli et al. [86]. Through competitive adsorption, BAs were able to displace other surface-active molecules from the LC/water interfacial adsorption. The competitive adsorption of bile acids and S_12_S on the surface of CLC droplets resulted in a change in droplet configuration from homeotropic to planar (Figure 4g). This innovative technique allowed for detecting BAs with general optical microscopes. This method had a detection limit of 1 μM.

### 3.2. Nucleic Acid

Nucleic acids, such as deoxyribonucleic acid (DNA) and ribonucleic acid (RNA), maintain genetic information for the next generation of organisms by storing it in nucleotides [87,88]. Therefore, DNA detecting is of great importance [89,90,91,92,93]. Recently, in DNA detection, LC droplet platforms have been extensively investigated by functionalized LC interfaces.

**Figure 5:**
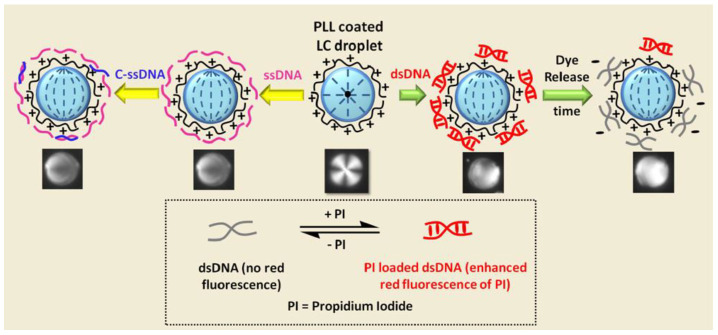
Schematic illustrations of poly(L-lysine)-coated LC droplets for sensitive detection of DNA and their applications in controlled release of drug molecules. Reprinted with permission from ref. [94]. Copyright 2017 American Chemical Society.

Verma et al. [94] developed a sensor based on LC droplets to detect DNA. Positively charged poly(L-lysine) (PLL) coated droplets could significantly absorb negatively charged DNA, which caused the molecules’ orientation of the droplets to change. They have also effectively shown that these DNA and PLL-decorated droplets exhibited the gradual release of propidium iodide (PI) dye from the DNA molecule during physiological circumstances (Figure 5).

Recently, DNA was used as a model biomarker to achieve excellent detection of biological molecules [95,96,97,98,99,100,101]. For continuous detection, Ma et al. [102] revealed a unique, simple, and ultra-low sample consumption assay method using WGM. To create a fiber optic probe, the sensor combines LC droplets and a hollow capillary tube (HCT). The test solution maintained a stable suspension of the LC droplets. The DNA detection was then accomplished by measuring the LC droplet orientation change and WGM spectroscopy. When used to identify the target salmon sperm DNA, it produced a measurement range that could be adjusted from 3.75 to 11.25 g/mL with a sensitivity of 0.33 nm/g/mL. The test solution only needed 3 nl of the sample, and the limit of detection was 1.32 g/mL, or as little as 3.96 pg of DNA could be effectively detected.

### 3.3. Protein and Peptides

All vital cellular and organismal functions, including immunological responses and cell communication, depend on proteins [103,104,105]. Numerous studies have been done on abnormal protein production in the early stages of illness [106,107]. Additionally, LC droplet-based biosensors for the detection of proteins and peptides have been created.

Bao et al. [108] described a novel biosensor based on phospholipid-coated NLC droplets and demonstrated the detection of Smp43, a model antimicrobial peptide (AMP) derived from scorpion venom. Monodisperse lipid-coated LC droplets of 16.7 ± 0.2 μm in diameter were generated using a PDMS microfluidic device and were targeted by AMP. Droplets were confined in a microfluidic trap and treated simultaneously with gradient concentrations of Smp43 in six different chambers. A significant change in the droplet appearance that corresponded with the transition demonstrated that the Smp43 (<6 μM) disrupts the lipid monolayer, at concentrations well within its physiologically active range. It had the potential to be developed into a trustworthy, affordable, and disposable point-of-care testing kit.

**Figure 6:**
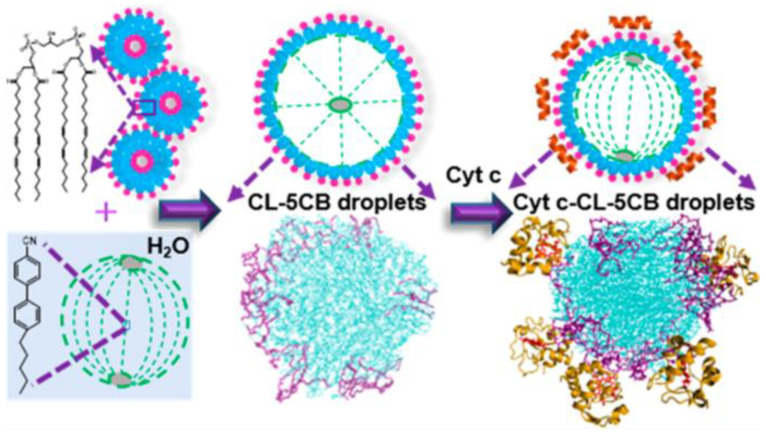
Schematic illustration of lipid-protein interactions that control the reorientation at the LC droplet interface. Reprinted with permission from ref. [109]. Copyright 2021 American Chemical Society.

To learn more about molecular interactions at the droplet interface, Pani et al. [109] employed aqueous LC dispersions to investigate the interaction between mitochondrial cardiolipin (CL) and membrane-associated cytochrome c (Cyt c) (the latter of which is essential for the apoptotic signaling cascade). The CL wrapped around the surface of 5CB droplets and bound to Cyt c at the droplet surface in an aqueous phase environment (Figure 6). The lipid-protein interactions causing the reorientation of the LC droplet interface are shown for the first time by integrated atomic calculations, microscopic readings, and spectroscopic observations.

The addition of proteins and peptides disrupts the arrangement of the self-assembled monolayer at the water/LC interface, leading to an orientation shift of LC molecules. On the surface of 5CB droplets, Bera et al. [110] adsorbed positively charged poly(diallyldimethylammonium chloride) (PDADMAC) and poly(ethylenimine) (PEI) of various molecular weights. The droplets treated with PDADMAC and PEI exhibited radial structure in aqueous solutions with salt contents greater than 150 mM. Electrostatic interactions caused the positively charged PDADMAC- and PEI-modified 5CB droplets to adsorb negatively charged bovine serum albumin (BSA), which then caused the droplets to change from radial to bipolar conformation. As PDAMAC and PEI molecular weights decreased, the BSA concentration necessary to cause the conformational change rises linearly.

By decorating PLL on LC droplets, Verma et al. [111] have described a simple but effective approach that permits label-free imaging of fibronectin (FibN) (major component of an extracellular matrix) adsorption at the LC/water surface. The PLL could induce vertical alignment of the LC at the LC/water interface so that PLL-adsorbed LC droplets displayed a radial configuration. Subsequent non-specific electrostatic adsorption on anionic proteins could trigger a rapid shift of the PLL-LC droplet director configuration to pre-radial or bipolar. This research developed an easy method based on LC droplets that may be useful in biological and interfacial systems. They [112] used the same method for real-time detection of the other proteins, such as BSA, concanavalin A (ConA), and cathepsin D (CathD). These proteins may be quantified with detection limits of 0.1, 50, and 250 g/mL for BSA, ConA, and CathD.

**Figure 7:**
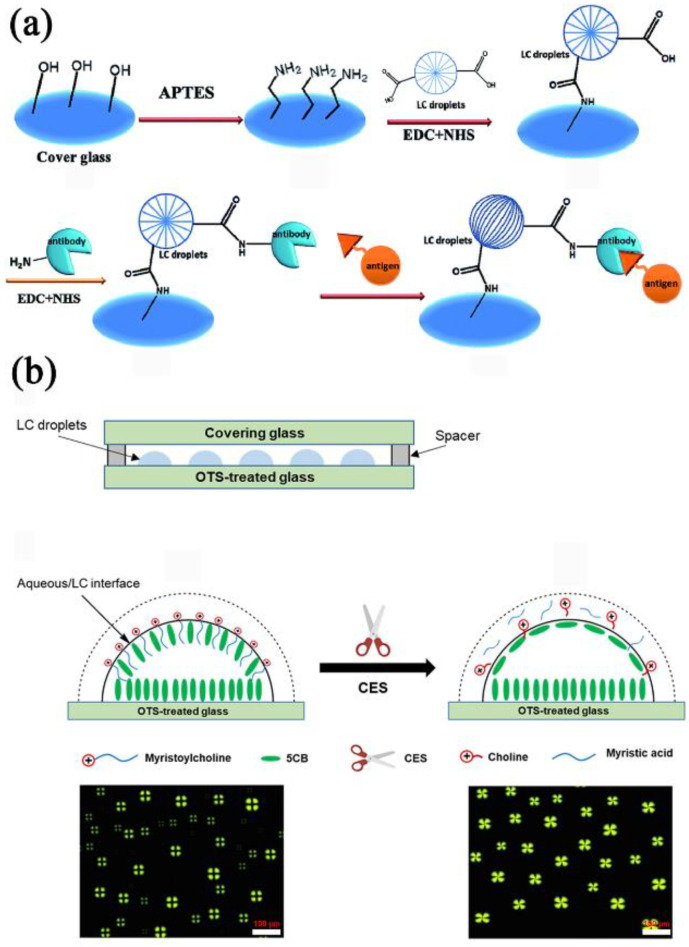
(**a**) Schematic illustration of slide cover glass immobilized LC microdroplets for sensitive detection of an IgG antigen. Reprinted with permission from ref. [117]. Copyright 2017 American Chemical Society. (**b**) Schematic illustration of using a LC droplet sensing platform to detect CES and its inhibitors Reprinted with permission from ref. [118]. Copyright 2022 MDPI. The scale bar is 100 µm.

The antibody–protein recognition reaction [113,114,115,116] at the LC droplet interface is also a detection method. Rabbit IgG antigen sensing LC droplets were created by Huan et al. [117]. Interfacial modifiers were poly(styrene-b-acrylic acid) copolymer (PS-*b*-PA) and SDS. Immobilized AIgG coupled LC droplets on glass slides were sensitive to optical signals interacting with IgG antigens in PBS and other media such as 10 wt% fetal bovine serum (FBS) and plasma (Figure 7a). Using slide cover slides, the detection limit of IgG antigen was reduced to 25ng mL^−1^ after contact with IgG antigen in PBS for 30 min at room temperature, which increased the possibility of effective interaction between IgG antigen and immobilized AIgG coupled LC droplets. The slide-coverglass-immobilized LC microdroplets have also demonstrated good archival durability and reusability in sensitive IgG detection.

Recently, Nguyen and Jang [118] developed a LC droplet biosensor for the detection of carboxylesterase (CES). They showed the sensor’s structure diagram and working principle (Figure 7b). After evaporating the heptane, a 1.5 μL heptane solution containing 2% (*v*/*v*) 5CB was coated on the OTS-modified glass slide to obtain the LC droplet pattern. The hydrophobic tail of the 5CB molecule was isotropic at the glass/LC interface. Using myristocholine (Myr) as a cationic surfactant, anterograde anchoring occurs at the LC/water interface, related to the dark cross morphology of LC droplets under POM. In addition, the effect of inhibitors on the activity of CES was observed. With good selectivity, the biosensor provided rapid and simple detection of CES and its inhibitors.

Block copolymers are widely used to design LC droplet biosensors to detect different targets. Polyelectrolytes (PEs) were functionalized on the surface of the already oppositely charged surfactant-coated 5CB droplets by Yang et al. [119], and the difference in their configurational orientations was aimed at detecting protein. The 5CB droplets coated with SDS and DTAB (5CB_SDS_ and 5CB_DTAB_) were functionalized by quaternized poly (4-vinylpyridine) (QP4VP) and polystyrene sulfonate (PSS). Hemoglobin and BSA were used as model proteins, and QP4VP-functionalized 5CB_SDS_ droplets were used for protein assays, then resulted in a radial-bipolar configurational shift and a decrease in charge density at LC/water interface. The demonstration of non-specific protein detection using the PE-functionalized 5CB droplets was then accomplished effectively.

**Figure 8:**
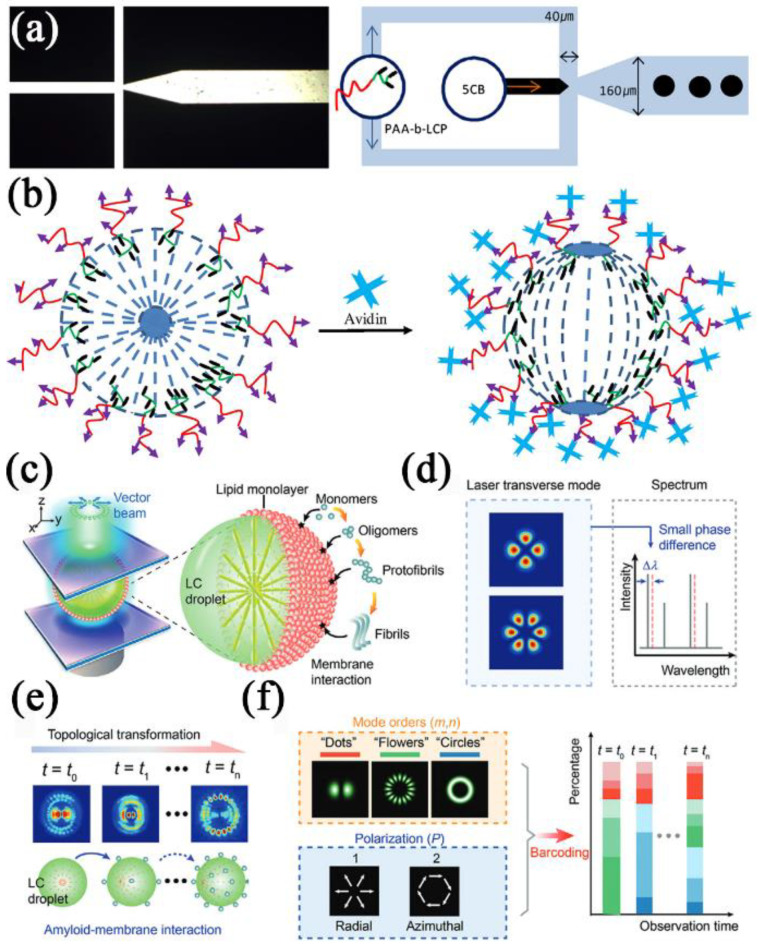
(**a**) An optical image (left) and a schematic (right) of the microfluidic channel with dimensions. (**b**) Schematic illustration of radial to bipolar transition of the 5CB_PAA_-biotin in an avidin aqueous solution. Reprinted with permission from ref. [121]. Copyright 2015 Elsevier. (**c**) Schematic illustration of generating a vector beam driven by molecular interaction. (**d**) Comparison of laser mode with conventional spectra interrogation. (**e**) Schematic illustration of topological transformation in laser mode pattern. (**f**) Illustration of the developed encoding rule. Reprinted with permission from ref. [126]. Copyright 2021 John Wiley and Sons.

According to Khan et al. [120], protein detection was investigated using poly(acrylic acid-b-4-cynobiphenyl-4′-undecyl acrylate) (PAA-*b*-LCP) coated LC droplets (LC_PAA_ droplets), in which the PAA chain attached the proteins at LC/water surface, and the LCP chain anchored LCs in the droplet. Using a POM and UV/Vis spectroscopy, they examined the binding of protein to the LC_PAA_ droplet. The unique radial-to-bipolar configuration shift caused by protein adsorption in the LC_PAA_ droplets was compared to the number of proteins adsorbed as determined by UV/Vis spectroscopy. After that, they [121] also used the PAA-*b*-LCP functionalized 5CB droplets by the microfluidic approach (Figure 8a). To identify avidin-biotin binding exclusively at the LC/water interface, the PAA blocks on 5CB droplets were biotinylated. As seen by POM, the shift in the structure of the 5CB droplet acted as a signal for the avidin-biotin binding (Figure 8b). With a detection limit of 0.5 μg/mL antibiotic protein, maximum biotinylation was accomplished by injecting >100 μg/mL of biotin aqueous solution.

Lasers are the backbone of modern photonics and sensing [122,123]. Micro lasers from biointegrated systems show clear advantages in biochemical analysis with improved sensitivity [124]. It has been recently applied to LC droplets. Wang et al. [125] described a LC droplet-based electrostatic responsive microlaser and examined its use for sensitively detecting negatively charged biomolecules. Four orders of magnitude improvements in sensitivity and dynamic range were made compared to a traditional POM method. The future uses of microlasers with a detection limit of 1 pM were lastly demonstrated using BSA and particular biosensing, providing new options for ultrasensitive label-free biosensing and monitoring of molecular interactions. Vector beams may be used to observe dynamic molecular interactions by following molecules’ topological properties, as Gong et al. studied [126]. The idea of amplified structured light molecular interactions was presented to track minute biological structural changes in microcavities. In a Fabry–Pérot cavity sandwiched between two biomimetic LC droplets, a modest protein-lipid membrane interaction caused the output vector beam to undergo a topological transition. It was discovered that varying molecule concentrations and sizes might cause the vector beam topology to change in real-time at various intervals (Figure 8c–f).

### 3.4. Cell and Microorganism

The targets of biosensors also include the pathogens that cause diseases in humans, such as cancerous cells, harmful bacteria, and viruses [127,128,129,130,131]. The main method of detecting cells and bacteria was achieved by the interaction between cell membranes and compounds that have a strong affinity for cells and bacteria [132,133]. Moreover, monitoring the role of substances in living cells is important for understanding the behavior and heterogeneity of tumor cells [134]. This section will discuss them in detail.

#### 3.4.1. Single-Cell Monitoring

Sidiq et al. [135] described a straightforward way to generate LC droplets via PLL-LC interactions in situ, which might be used to report the existence of cells and to track how those cells interacted with their environs in real-time via topological flaws in those droplets. Additionally, it has been shown that responsive PLL droplets might be used as a template for reporting Annexin V-phosphatidylserine interactions.

**Figure 9:**
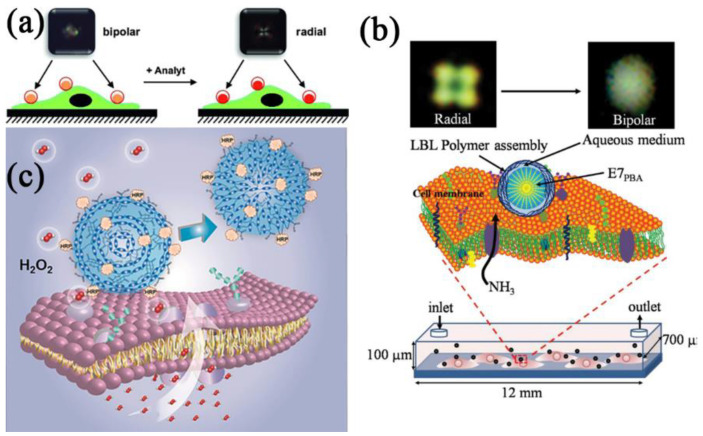
(**a**) Schematic illustration of LC droplet-based chemical sensors. Reprinted with permission from ref. [136]. Copyright 2013 John Wiley and Sons. (**b**) Schematic illustration of immobilized P-E7_PBA_ droplets on cells cultured in a microfluidic channel. NH_3_ released from the cell results in a radial-to-bipolar change of the E7_PBA_ encapsulated in the polymeric microcapsule. Reprinted with permission from ref. [138]. Copyright 2019 John Wiley and Sons. (**c**) Schematic illustration of LCEM-HRP immobilized on the cell membrane and its reversible transfiguration. Reprinted with permission from ref. [139]. Copyright 2020 John Wiley and Sons.

The creation of LC droplet-based sensors that might be fixed directly on the surfaces of cells was described by Manna et al. [136]. The E7 was injected into covalently cross-linked microcapsules made of reactive layers of polyethyleneimine (PEI) and poly(4,4-dimethyl lactone) (PVDMA) to create LC droplets. The sensors might report the presence of dangerous compounds in the immediate environment at the level of individual cells and single droplets (Figure 9a). The thermotropic LC droplets, as tiny as a micron in size, might be inserted inside living human cells and employed as chemical sensors to find poisons in extracellular situations, according to their further research [137].

According to Khan et al. [138], the LC E7 was filled with polymeric microcapsules after being decorated with 4-pentylbipenyl-4-carboxylic acid (PBA) (P-E7_PBA_). The P-E7_PBA_ droplets were immobilized on cells grown in a microfluidic channel. Live imaging of NH_3_ produced from the cells or a single cell was made possible by changing the orientation of P-E7_PBA_ from radial to bipolar during cross-polarization (Figure 9b). The P-E7_PBA_ offered the benefits of a regulated size to avoid endocytosis, simple cell membrane immobilization, selective NH_3_ release detection, high sensitivity, and simple POM detection.

Recently, Li et al. [139] initially created chemically responsive LC elastomer microspheres (LCEM), which were functionalized by horseradish peroxidase (LCEM-HRP), to observe the release of H_2_O_2_ from a single live cell in real-time (Figure 9c). They looked at the release of H_2_O_2_ from normal human umbilical vein endothelial cells (HUVEC), human primary glioblastoma (U87), lung cancer cells (A549), and hepatocellular liver carcinoma cells (HepG-2). It was shown that each LCEM-HRP could transmit H_2_O_2_ in real-time with single-cell resolution using a concentric-to-radial (C-R) transfiguration. The system’s inter- or intra-chain hydrogen bonding was broken, causing the C-R transfiguration caused by the system’s decrease of H_2_O_2_ by HRP.

#### 3.4.2. Different Cells and Microorganisms’ Detection

Tumor cells are an important biomarker, and their detection has important clinical significance [140,141,142,143,144]. Yoon et al. [145] demonstrated a cell-selective LC droplet emulsion using folic acid-conjugated block copolymers(PS-*b*-PAA-FA) and sodium dodecyl sulfate (SDS) as a mediator. The created LC droplet emulsion showed a configurational shift from radial to bipolar when engaged with KB cancer cells. Still, no such change was seen when the emulsion was permitted to touch normal cells, for instance, fibroblast and osteoblast. After that, the same group [146] presented β-galactose-conjugated poly(styrene-b-acrylic acid) block copolymer (PS-*b*-PA-G) for an LC microdroplet-based sensing system utilized 5CB. Interaction of HepG2 cells with PS-*b*-PA-G induced a radial to bipolar orientation shift of liquid crystal microdroplets.

With the rapid development of biosensing technology, many methods for detecting bacteria have emerged [147,148,149,150,151]. Traditional approaches are time- and money-consuming, despite effectively detecting different cancer cells with high sensitivity and accuracy. Sivakumar et al. [70] reported monodisperse LC droplets as a versatile sensing method that could distinguish various bacterial and viral strains (Gram +ve and −ve). Filling the PEM capsules formed from PMA/PVPON with 5CB-generated LC droplets. When in contact with Gram −ve bacteria (*E. coli*) and lipid enveloped viruses (A/NWS/Tokyo/67), the transition of LC droplets from bipolar to radial occurred. Small amounts (1–5) of *E. coli* bacteria and low concentrations (10^4^ pfu/mL) of A/NWS/Tokyo/67 viruses can be detected using the sensor.

**Figure 10:**
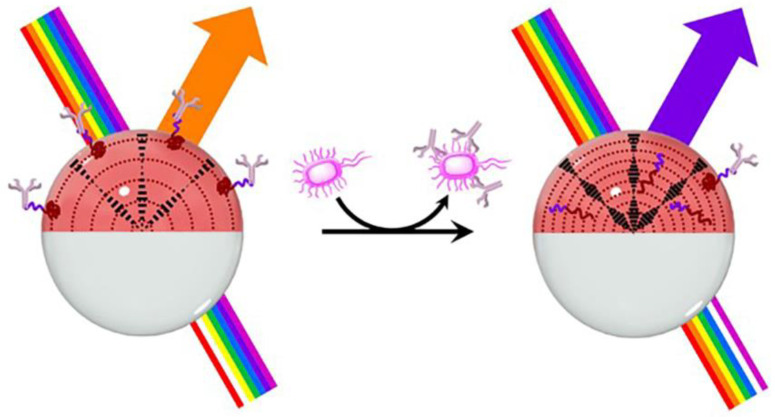
Schematic illustration of the mechanism for the detection of *Salmonella enterica* using chiral nematic (N*) complex emulsions. Changes in the reflected light are produced through changes in the interfacial activity of boronic acid polymeric surfactants induced by a competitive binding/unbinding of IgG antibodies at the LC/W interface. Reprinted with permission from ref. [152]. Copyright 2021 American Chemical Society.

Recently, Concellón et al. [152] described how to create a novel optical sensor system using complicated N* LC emulsions (Figure 10). The emulsions had two immiscible compartments made of an N* LC and a fluorocarbon oil, and they might be dynamically reconfigured. The N* pitch dynamically changed in response to the presence of microorganisms. This pitch modulation was accomplished by utilizing chiral polymer surfactants with boronic acid capabilities. As a result of the manipulation of the chiral polymer at the LC/water interface predicted that the antibody’s interaction with the target bacterium would result in optical changes. They showed that these alterations result in optically readable and triggered reflectance alterations that might be employed as a reliable optical read-out for identifying the foodborne pathogen *Salmonella*.

### 3.5. Drug

Although drugs can be used to treat illnesses, they can also damage living things if taken in excess [153,154]. Detecting the content of drugs is crucial [155,156,157,158,159]. *Streptomyces kanamyceticus* produces the aminoglycoside antibiotic known as kanamycin, which is prescribed to animals to treat infections. Kanamycin may build up excessively in the human body, causing antibiotic resistance as well as adverse consequences such as ototoxicity and nephrotoxicity that might ultimately result in catastrophic harm to the body [160]. Therefore, it is essential to find kanamycin residues. The need for straightforward, practical, affordable methods for quick and label-free identification is growing. Yin et al. [161] reported a novel idea for the surface-anchored LC droplet-based detection of kanamycin. With a rise in surfactant concentration, the optical pictures of the LC droplets progressively transition from a four-leaf clover appearance to a uniformly dark cross appearance. A kanamycin aptamer and the cationic surfactant cetyltrimethylammonium bromide (CTAB) were used to detect kanamycin. The addition of the aqueous solutions of CTAB and CTAB/aptamer complex caused the LC droplets to look uniformly black and four-clover-shaped, respectively. However, the CTAB could be released by the precise binding of kanamycin to its aptamer, leading to the uniformly black appearance of the LC droplets. A portable instrument was created to test the optical brightness of the LC droplets. This technique enabled the detection of kanamycin in actual samples like milk and honey as well as at concentrations as low as 0.1 ng/mL (or 0.17 nM). A portable optical device helped the development of novel LC-based sensor types using surface-anchored LC droplets.

Myricetin (MY) is effective in scavenging free radicals. Myricetin may serve as an anti-tumor agent by promoting the breakdown of double-stranded DNA when it interacts with DNA [162]. The rapid dosage configuration of MY is crucial for its application in a variety of therapeutic therapies for different malignancies [163,164]. Detection of MY has been suggested and demonstrated using a 5CB droplet-based sensing technique by Xiong et al. [165]. It was created using a syringe pump attached to a tapered capillary microtube, and it was functionalized in the aqueous phase using DTAB and DNA. When the MY concentration increased, the 5CB microdroplet showed a structural shift. Additionally, they created typical WGM lasing spectra by employing the 5CB microdroplet as an optical microcavity of the WGM. The WGM spectrum showed a spectral blue shift with an increase in MY addition. The sensitivity was 0.04 nm/μM when used within the detection limit.

### 3.6. Toxic Chemical

#### 3.6.1. Toxin

Endotoxin, a component of the cell wall of Gram -ve bacteria, consisted of two polysaccharide domains and a glycosphingolipid (lipid A) [166,167]. Jiang et al. [168]. reported that using experimental measurements based on NLC droplets and machine learning techniques, bacterial sources can be classified, and the concentration of endotoxin derived from three bacteria presented in an aqueous solution could be quantified. They showed how EndoNet was used to detect subtle changes in the scattering field. This allowed them to classify bacterial sources and measure endotoxin levels in an eight-order magnitude range from 0.01 pg mL^−1^ to 1 g mL^−1^.

**Figure 11:**
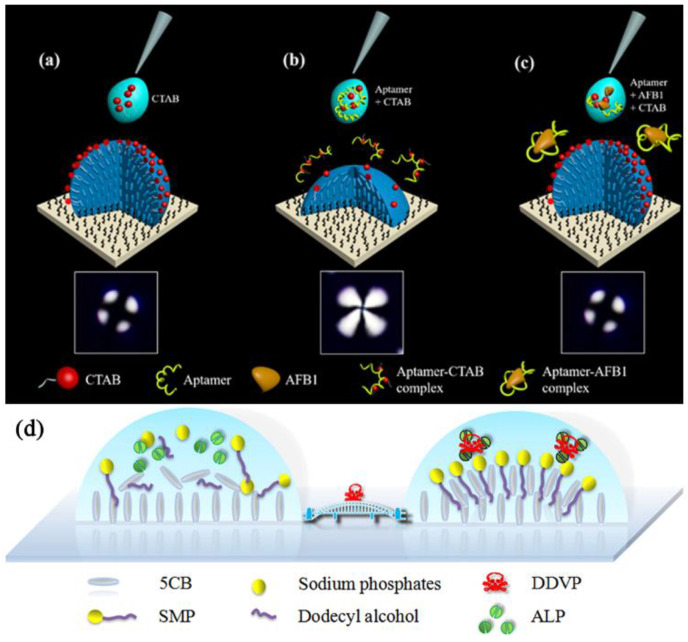
Schematic diagram of the experimental principle. The POM images of the 5CB_SADrop_ and their corresponding configurations in the aqueous solutions of (**a**) CTAB, (**b**) CTAB and AFB1 aptamer, and (**c**) CTAB, AFB1 aptamer, and AFB1, respectively. The dark cross appearance and the four-leaf clover appearance correspond to the radial and escape-radial configurations of LC droplets, respectively. Reprinted with permission from ref. [172]. Copyright 2022 Elsevier. (**d**) Schematic illustration of orientation states for LCs: the absence and presence of DDVP in ALP solution on the SMP doped-5CB droplet patterns. Reprinted with permission from ref. [178]. Copyright 2018 Elsevier.

Aflatoxin is the most toxic and carcinogenic mycotoxin. Consumption of Aflatoxin B1 (AFB1)-contaminated food, even in small amounts, might lead to cumulative effects and pose a risk to human health [169]. Therefore, to ensure food safety against mycotoxins, highly sensitive and reliable detection methods are needed to investigate the causes of food poisoning [170,171]. Recently, Cheng et al. [172] showed how to employ surface-anchored 5CB droplets (5CB_SADrop_) on a N, N-dimethyl-N-octadecyl(3-aminopropyl)trimethoxysilyl chloride (DMOAP)-coated glass to provide a straightforward and practical method for detecting AFB1 in food samples. The evaporation of a solvent was used to create the surface-anchored 5CB droplets (5CB_SADrop_). Figure 11a–c depicted the experimental design’s basic principles. A black cross appearance matching a radial orientation of the LC droplets was seen under the POM (Figure 11a) when the surfactant CTAB was put onto the 5CB_SADrop_. The escape-radial shape of the LC droplets in a combination of CTAB and AFB1 aptamer resembled a four-leaf clover in the look of the 5CB_SADrop_, in contrast (Figure 11b). In addition, when a combination of CTAB, AFB1, and its aptamer was dropped on the 5CB_SADrop_, a radial structure of the particle was seen (Figure 11c). Through the appearance of 5CB_SADrop_ under the POM, the various AFB1 concentrations were identified. Additionally, peanut oil and rice samples were used to demonstrated AFB1 could be detected practically. The 5CB_SADrop_ technique was particularly promising for use in the food and agricultural industries since it possessed the advantages of simple analysis, minimal sample consumption (1 μL), significant sensitivity, excellent stability, quick on-field detection, and removal of the need for tags and pricey apparatus, and low cost.

#### 3.6.2. Pesticide and Pollutant

The environmental problems of the earth are becoming more and more serious. It is very important to detect the content of environmental pollutants [173,174,175,176,177]. Based on enzymatic reactions, LC molecules can be employed to detect pesticides, as was stated for enzyme detection. The responsiveness of microdroplets floating in aqueous environments made the creation of new types of LC droplet environmental sensors. Zhou et al. [178] built straightforward yet reliable 5CB droplet sensors for accurate and practical dichlorvos (DDVP) detection based on its hydrolysis by alkaline phosphatase (ALP). Using sodium monododecyl phosphate (SMP), an ALP cleavable surfactant, to change the orientations of 5CB, LCs were able to regulate their optical responses (Figure 11d). Because of the development of the SMP monolayer at the water/LC droplet interface, a dark crossed optical image of 5CB was obtained. After adding the combination of ALP and SMP, the optical appearance of 5CB becomes a dazzling fan-shaped appearance. Interestingly, the combination of pre-incubated DDVP and ALP LCs retained dark optical images under POM. The detection limit of DDVP was 0.1 ng/mL.

Volatile organic chemicals (VOCs) are often utilized in chemical synthesis as precursors. The majority of VOCs are toxic and combustible, which raises serious questions about their safety for both people and the environment [179]. Numerous illnesses, such as lung cancer and chronic respiratory inflammation, could be brought on by prolonged exposure to these vapors [180,181,182,183]. The LC droplets could be a promising platform to detect various VOCs. For instance, An et al. [184] developed a sensing method based on the LC droplet pattern method to detect and monitor small amounts of organic aldehyde fumes. Exposure of LC droplet pattern coated in glycine solution to aldehyde fumes causes the light signal to shift from a bright sector to a dark cross appearance, as demonstrated by a POM. The results revealed the glycine/LC droplet pattern technology’s excellent sensitivity and selectivity. The signal change was completed in a couple of minutes when the sensor was exposed to the aldehyde in a realistic environment (2–7 min).

Recently, deep convolutional neural networks (CNN) were investigated by Frazo et al. [185] for using as pattern recognition systems to examine the dynamics of optical textures in LC droplets subjected to various VOCs. A single droplet was shown to discriminate among 11 types of VOCs with slight structural and functional differences. Regression modeling led to the hypothesis that fluctuations in a droplet’s optical texture pattern also represented changes in VOC content. As a result, the CNN-based methodology offered a possible means of detecting VOCs from the reactions of individual LC-droplets.

### 3.7. Other Molecules

Aside from the biomolecules just listed, some other molecules are also used as targets for biosensors [186,187,188,189,190]. Liquid crystal biosensors have also been created to identify additional substances including urea, glucose, and so on. These LC sensors for detecting more biological molecules are discussed in this section.

**Figure 12:**
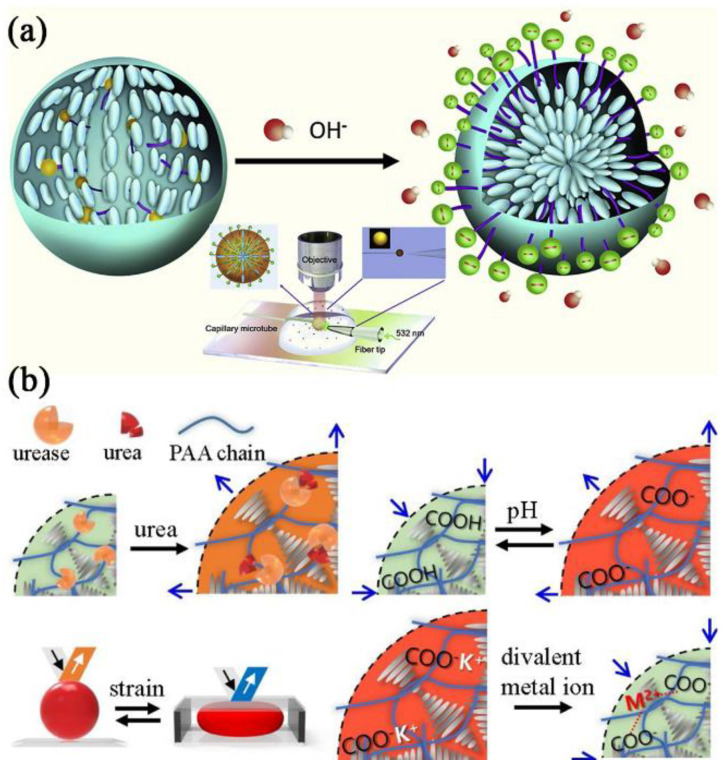
(**a**) Schematic illustration of the structural transition of stearic acid-doped 5CB microdroplet from planar anchoring to homeotropic anchoring. Reprinted with permission from ref. [41]. Copyright 2019 Elsevier. (**b**) Several tens of micrometer-sized photonic solid-state cholesteric LC (CLCsolid) balls have been functionalized with a weak anionic polyelectrolyte of poly(acrylic acid) in the form of an interpenetrating polymer network (IPN). Reprinted with permission from ref. [194]. Copyright 2020 Elsevier.

Urea serves as a crucial biomarker for the detection and clinical evaluation of urological illnesses [191,192]. Human urine typically has urea concentrations between 155 and 390 mM. The urea content in urine, however, can go over this range when renal function is compromised or when the glomerulus’s effective filtration area is diminished [193]. To detect urea, Duan et al. [41] employed stearic acid as a functional material and used urease’s highly effective urea enzymolysis. In the reaction with urea and urease, the pH of the surrounding environment increases, and the stearic acid undergoes deprotonation at the aqueous/LC interface, which caused the formation of 5CB molecules to change (Figure 12a). In the interim, the LC microdroplet’s director configuration changed appropriately from bipolar to radial. In this work, the lasing mechanism was unmistakably established by thorough spectroscopic research and assigned to WGMs. The sensor could detect urea molecules at a concentration of only 0.1 mM. A strategy for creating complete solid-state CLC balls (CLC_solid_) with a semi interpenetrating polymer network (IPN) structure (CLC_solid-IPN_) has been reported by Lim et al. [194]. The suggested technique entailed the creation of pH-sensitive CLC_solid-IPN_ microspheres, their subsequent functionalization, which included the immobilization of receptors for the biosensor platform, and the fabrication with perfect spherical symmetry and uniformed concentric layer thickness (Figure 12b). The generated photonic CLC_solid-IPN_ microspheres were functionalized and analytically tested in the chamber of a specially designed PDMS chip. After immobilizing urease as a biosensor and applying KOH treatment, the effectiveness of the urea and heavy metal ion detection tests was demonstrated using the PDMS sensor chip with photonic CLC_solid-IPN_ microspheres (Figure 12b).

Glucose is essential to the health of the body because it serves as the primary energy source for cells and other living things [195]. The oxidation of glucose in the presence of glucose oxidase (GOx) is the most common mechanism for glucose detection [196,197,198]. Using a microfluidic device, Lee et al. [199] generated CLC microspheres of uniform size and decorated them with PAA-*b*-LCP (called CLC_PAA_ microspheres). Then, the GOx enzyme was immobilized on CLC_PAA_ microspheres with high or low chiral dopant contents and alterations in their helical shapes and coloring patterns took place in the presence of glucose. The CLC_PAA_ microspheres immobilized with GOx (CLC_PAA-GOx_) demonstrated great sensitivity in detecting glucose (0.5 μM) and quick reaction (≤4 s). Liquid crystal droplet-based non-enzymatic glucose sensing has also been reported. Munir and Park [200] described 5CB microdroplets coated with 3-aminophenyl boronic acid (APBA), which had an excellent sensitivity and specificity for the detection of glucose. The LC droplet biosensor for glucose detection showed excellent stability over 30d, robust selectivity against cholesterol, uric acid, and acetaminophen, and exceptional sensitivity even in complex blood samples (detection limit of 0.05 mM). This glucose LC biosensor had the potential to take place of enzyme-based ones since it was more reasonably priced and reliable. In conclusion, LC droplet-based sensors will continue to dominate the area of glucose sensing.

**Figure 13:**
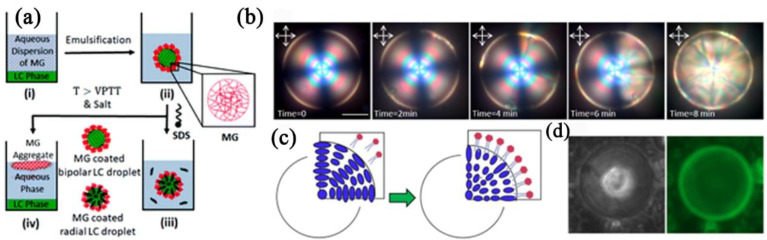
(**a**) Schematic illustration of essential steps for emulsification of (i) two-phase system, (ii) formation of an aqueous dispersion of MG stabilized LC droplets, (iii) response of LC droplets to SDS, and (iv) on-demand emulsion breaking. Reconfiguration of high-chirality LC droplets in the presence of DLPC amphiphiles. Reprinted with permission from ref. [201]. Copyright 2019 Royal Society of Chemistry. (**b**) Reflection mode POM images of reconfiguration dynamics in 30 µm high-chirality droplets in the presence of 0.5 mM DLPC. (**c**) Schematic of the planar to homeotropic ordering transition. (**d**) Bright-field and fluorescent image of adsorbed labeled DLPC amphiphiles on the chiral droplet’s interface. Reprinted with permission from ref. [202]. Copyright 2022 MDPI. The scale bar is 20 µm.

Additionally, in a novel approach, Dan et al. [201] reported that LC droplets adorned with microgels (MGs) exhibited extraordinary stability (Figure 13a). This technology made it easier to analyze LC droplets that underwent a conformational shift caused by analytes (such as SDS), which improved the quantitation of aqueous analytes.

The interaction between biomolecules and CLC was recently studied by Norouzi et al. [202]. They looked at how the structure of CLC molecules in droplets was affected by the phospholipid 1,2-diauroyl-sn-glycero3phosphatidylcholine (DLPC). The influence of droplet size and DLPC concentration on the structural remodeling of the CLC molecules was seen (Figure 13b). Their results showed that the CLC droplets transition from planar to homeotropic ordering due to a multistage molecular reorientation in the presence of DLPC (Figure 13c,d). However, this reconstruction process was carried out three times faster in low chiral droplets than in high chiral droplets.

## 4. Conclusions and Future Directions

In this review, we summarized the advancement in LC droplet-based biosensing techniques for biomolecular detection. Liquid crystal has become one of the best materials for biosensing technology. The LC droplet sensors are used in the field of bioanalysis and have gained popularity due to their advantages such as easy availability, low sample consumption, simple operation of detection instruments, and low cost. Additionally, the LC droplet biosensors with the optical signal response compete in terms of sensitivity with other detection techniques [203,204].

Due to their excellent mobility and endurance, LC droplet-based biosensors can potentially be used for the POC diagnosis of various diseases. It is ideal to build user-friendly LC droplet-based sensors using integrated devices. For example, microfluidic devices and capillary-based devices can be designed in combination with LC droplet as POC devices. The development of a new generation of high-performance and portable LC droplet sensors has become popular due to the continued advancement of science and technology as well as the interpenetration and intersection of disciplines. These sensors can achieve more accurate and quick detection and analysis of biomolecules and are also suited for widespread application. Therefore, these easy-to-use methods have the potential to replace complicated, specialized detection techniques. Recently, the application of LC droplet sensors to single cell analysis has gradually become a novel research direction. Liquid crystal droplets immobilized on cell membranes can image the metabolite release from living cells. The LC droplet-based sensing devices have the potential to be designed as efficient and ultra-sensitive imaging systems.

Although LC droplet biosensors have many advantages, it should be recognized that they also have shortcomings. Typically, LC droplet biosensors detect only one target, so designing them for simultaneous detection of multiple targets remains limited. And the performance of these sensors can be damaged at high temperatures because LCs lose contrast under such conditions. Usually, most of the LC droplet-based biosensors are designed for visualization using POMs. We believe that other better signal readout methods can be developed, leading to the development of superior and more sensitive LC biosensors.

In the future, we expect that researchers will overcome the difficulties and develop LC droplet-based biosensors that are simpler to operate, more responsive, and more stable. Furthermore, we hope that these sensors can be used for trace biomarker detection for home and field diagnostics by general users far from central laboratories.

To fulfill the present demands for trace detection of biomolecules, further research of LC droplet sensors is being pursued from the following perspectives:

1. Spherical LC microstructures with one or more stable cores and multiple nesting may be created because of the rapid advancement of microfluidic technology. The designability and diversity of complex “core-shell microstructures,” as opposed to simple LC droplets and shells, will offer them new features, and raise new scientific challenges that call for more in-depth investigation.
2. The pointing vector configuration and defects in solvated LC droplets and shells and the related photonics applications are also fascinating research areas that need further exploration.
